# Viral manipulation of the cellular sumoylation machinery

**DOI:** 10.1186/s12964-017-0183-0

**Published:** 2017-07-14

**Authors:** Angela J. Lowrey, Wyatt Cramblet, Gretchen L. Bentz

**Affiliations:** Division of Biomedical Sciences, Mercer University School of Medicine, Macon, Georgia

**Keywords:** Viruses, Small ubiquitin-like modifier, Sumo, Ubc9, Senp, Pias, RanBP2, SAE1, SAE2

## Abstract

Viruses exploit various cellular processes for their own benefit, including counteracting anti-viral responses and regulating viral replication and propagation. In the past 20 years, protein sumoylation has emerged as an important post-translational modification that is manipulated by viruses to modulate anti-viral responses, viral replication, and viral pathogenesis. The process of sumoylation is a multi-step cascade where a small ubiquitin-like modifier (SUMO) is covalently attached to a conserved ΨKxD/E motif within a target protein, altering the function of the modified protein. Here we review how viruses manipulate the cellular machinery at each step of the sumoylation process to favor viral survival and pathogenesis.

## Background

Post-translational modification of proteins is important to numerous cellular events, allowing cells to respond to both external and internal stimuli. The most understood modifications include ubiquitination, phosphorylation, acetylation, methylation, and glycosylation. In 1997, a new type of modifying protein (small ubiquitin-like modifier or SUMO) was identified [1]. Since then, four SUMO isoforms (SUMO-1, −2, −3, and −4) have been characterized in humans. Sequence alignment revealed that SUMO-2 and SUMO-3 are approximately 97% similar, so they are often referred to as SUMO-2/3. SUMO-4 shares around 86% identity with SUMO-2/3, while SUMO-1 has approximately 46% identity with SUMO-2/3. SUMO-1 and SUMO-2/3 are ubiquitously expressed in the body; however, SUMO-4 has been detected only in the kidney, dendritic cells, and macrophages [2]. Each SUMO can be covalently conjugated to the lysine residue found within the conserved ΨKxD/E motif, where Ψ represents a hydrophobic residue of the target protein, resulting in its sumoylation [3].

**Fig. 1:**
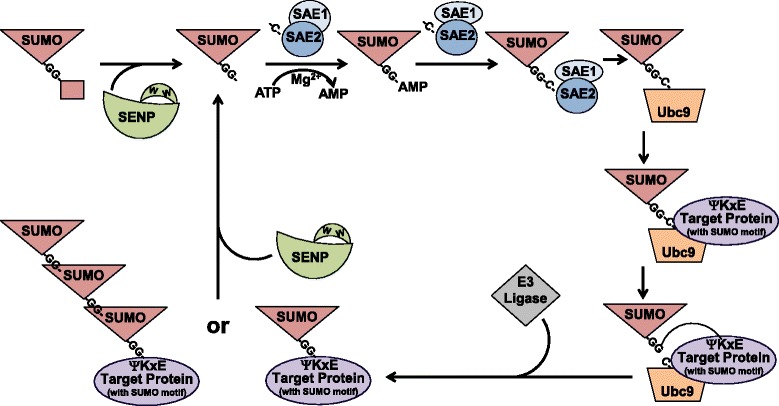
The Sumoylation Process. The small ubiquitin-like modifier (SUMO) pro-peptide is processed by the Sentrin-specific proteases (SENPs), during maturation, to reveal the C-terminal di-glycine motif. The SUMO-activating enzyme (composed of SAE1 and SAE2) adenylates the SUMO di-glycine motif in an ATP- and Mg^2+^-dependent manner. A transient intermediate thioester bond forms between SUMO and SAE2 C173. SAE2 passes SUMO to the ubiquitin-like conjugating enzyme (Ubc9), forming a second transient intermediate thioester bond. Ubc9 recognizes the ΨKxD/E SUMO motif within a target protein and catalyzes the formation of an isopeptide bond with the C-terminal SUMO di-glycine motif and the **ε**-amino group of the lysine residue within the SUMO motif of the target protein. The end result (sometimes with the assistance of a SUMO E3 ligase) is the mono- or poly-sumoylation of the target protein. The whole process can be reversed by SENPs, which contains a tryptophan tunnel that allows for the accurate positioning of the SUMO di-glycine motif and the cleavage of the SUMO-target protein isopeptide bond

The sumoylation process begins with the transcription and translation of the *sumo* genes to yield the SUMO pro-peptide (Fig. 1). A SUMO protease (see below) removes a small number of amino acids from the C-terminus of the pro-peptide to reveal the SUMO C-terminal di-glycine motif. The end result is the mature form of SUMO, which can be used to modify a target protein (Fig. 1). Mature SUMO is activated by the SUMO-activating enzyme, which is a heterodimer consisting of two subunits (SAE1 and SAE2, Fig. 1). Using ATP as a donor/substrate, SUMO E1 catalyzes the adenylation of the di-glycine motif of the mature SUMO, forming a SUMO-AMP intermediate. During this step, the SUMO E1 undergoes a conformational change, which then allows for the formation of a transient intermediate thioester bond between SUMO and a cysteine residue on SAE2 (C173).

Following activation, SUMO is passed to the SUMO-conjugating enzyme Ubc9 (Fig. 1), a 158-aa protein that forms a single domain structure similar to other ubiquitin conjugating proteins [4]. Ubc9 consists of four core β-sheets that are surrounded at the ends by four α-helices [4]. Within the pocket formed by these structures is the conserved catalytic cysteine residue of Ubc9 (C93). SAE1/2 transfers the SUMO to Ubc9 C93, forming a second transient intermediate thioester bond [5]. The Ubc9 pocket also identifies the canonical ΨKxD/E motif within the target protein (Fig. 1) [6]. The catalytic site of Ubc9 catalyzes formation of an isopeptide bond with the C-terminal SUMO di-glycine motif and the ε-amino group of the lysine residue within the SUMO motif of the target protein (Fig. 1) [6]. In addition to the interaction of SUMO with the pocket of Ubc9, the target protein interacts through non-covalent interactions with the surface of Ubc9. This surface is composed of numerous patches with positive and hydrophobic residues [4]. While Ubc9 has several SUMO-independent functions, it is proposed that the non-covalent SUMO-Ubc9 interactions enable the formation of poly-SUMO chains [7].

In some cases, the attachment of SUMO to the target protein also requires a SUMO ligase (E3) (Fig. 1), such as Ran binding protein (RanBP2) [8], a member of the protein inhibitor of activated STAT (PIAS) protein family [9], or the polycomb protein Pc2 [10]. These SUMO E3 ligases confer specificity towards the target protein and may help mediate the sumoylation of target proteins, including residues outside of the canonical ΨKxD/E motif. The SUMO E3 ligases are thought to interact with SUMO and Ubc9 and serve as adaptors between the Ubc9-SUMO intermediate and the target protein [11].

The entire process can be reversed by SUMO proteases or Sentrin-specific proteases (SENPs) (Fig. 1). In mammals, six SENP isoforms (SENP 1–3 and 5–7) with de-sumoylating activity have been identified [12]. These isoforms are divided into sub-families based on their cellular distribution, role in maturation of the SUMO pro-peptides, and/or their specificity in cleavage of SUMO-1- or SUMO-2/3-modified proteins. SENP1 and SENP2 make up the first sub-family due to their ability to cleave SUMO-1, −2, and −3 [12]. The second and third sub-families are SENP3 and SENP5 or SENP6 and SENP7, respectively, which preferentially cleave SUMO-2/3-modified proteins over SUMO-1-modified proteins [12]. In addition to de-conjugating sumoylated proteins, SENP1, SENP2, and SENP5, are also responsible for the maturation of the SUMO pro-peptides (Fig. 1) [12].

The SENPs share a conserved C-terminal cysteine protease catalytic domain [12], which has the typical catalytic triad (cysteine-histidine-aspartic acid). The C-terminal domain is formed by anti-parallel five-stranded β-sheets surrounded by two α-helices [13]. This structure interfaces with SUMO, allowing for the interaction between the SENP and SUMO precursors or sumoylated proteins [13]. Within the catalytic site, tryptophan residues form a tunnel that allow for the accurate position of the SUMO di-glycine motif and scissile bond [14]. Within the tunnel, the scissile bond creates a kink in the isopeptide linkage or within the SUMO pro-peptide and promotes the cleavage of the bond, resulting in protein de-sumoylation or SUMO maturation, respectively [15].

In a manner similar to ubiquitination, proteins can be mono- or poly-sumoylated. SUMO-2/3 contains the canonical SUMO motif, granting mature SUMO-2/3 the ability to sumoylated, forming poly-SUMO chains on target proteins [16]. However, SUMO-1 lacks this motif, so it can only be used to mono-sumoylate a target protein or act as a terminator of a SUMO-2/3 chain [17]. Little is known of how these forms differ in regulating protein function [3].

While only 5–10% of a target protein is found in a sumoylated form at any given time, the effect sumoylation has on protein function can be long-lived, even affecting protein function after it is de-sumoylated [18]. Sumoylation can regulate protein activity through altering a protein’s intracellular location, affecting a protein’s ability to interact with other proteins, and modifying a protein’s ability to interact with DNA [3, 19, 20]. Protein sumoylation also modulates cellular processes, including nuclear trafficking, cell division, DNA replication, DNA damage responses, transcription, and chromosome segregation [21]. Because of the multitude of cellular processes affected by protein sumoylation, dysregulation of sumoylation processes can significantly alter normal cellular events, such as cell motility and survival, and result in extremes, including cancer progression and viral pathogenesis.

### Viruses and sumoylation processes

While the intracellular pool of free SUMO-1 is considered to be limited due to its conjugation to high-affinity targets such as RanGap1 [22], SUMO-2/3-mediated sumoylation is primarily inducible by stress [23]. Global changes in protein sumoylation (by SUMO-1 and/or SUMO-2/3) occur following heat shock [23, 24], DNA damage [25, 26], and inhibition of the proteasome [27, 28], and other cellular stimuli, such as viral infection. During infection and replication, viruses can manipulate the sumoylation process to ensure viral persistence within the host. In addition, protein sumoylation has a role in mediating the antiviral effect of the interferons [29]. Through inhibition or induction of protein sumoylation, viruses have a multitude of mechanisms by which they manipulate this cellular process to ensure their survival and propagation.

Numerous viruses benefit from impaired sumoylation processes. Decreased sumoylation of specific antiviral proteins (promyelocytic leukemia protein/PML and Sp100) has been suggested to be important in regulating anti-viral immune responses. For example, herpes simplex virus-1 (HSV-1) infection results in a three-fold decrease in the modification of over 100 cellular proteins, including PML and Sp100, by SUMO-2/3 (Table 1) [30]. The observed changes were dependent on the viral ubiquitin ligase ICP0, which targeted the SUMO-2/3-modified proteins to the proteasome for degradation [31]. Many of the ICP0-targeted proteins are involved in the regulation of transcription, chromatin assembly, and chromatin modification, which suggests the importance of decreased protein sumoylation for lytic HSV replication [30]. Similarly, the Epstein-Barr virus (EBV) protein kinase BGLF4 suppresses global cellular sumoylation processes in order to facilitate EBV lytic replication [32], which suggests inhibition of sumoylation processes aids herpesvirus propagation.

**Table 1:** Viral targeting of the sumoylation machinery

	Global Sumoylation	*S*UMO levels	SAE1/SAE2	Ubc9	E3 Ligase			SENPs
					PIAS	RanBP2	Mimics	
Adeno			CELO Gam1 [41, 42]	CELO Gam1 [42]; ADV E1A [46]	ADV E4-ORF3: PIAS3 [64]		ADV E1B-55K [83]; ADV E4-ORF3 [110]	ADV pro.? [119]
Hepadna								
Herpes	Lytic HSV: decreased [30, 31]			HSV [65]	HSV ICP0: PIAS1/3/4 [60, 65, 66]	HSV [70]		
				HCMV IE1/2 [50, 54]; HHV-6 IE1/2 [57]	HCMV IE2: PIAS1 [50]	HHV-6? [78–80]		
	Latent EBV: increased [35]; Lytic EBV: decreased [32]	EBV LMP1?		EBV/KSHV ZTA/RTA [48, 51–53, 55, 56]; EBV LMP1 [35]			KSHV bKZIP [82]; KSHV LANA [56]	EBV LMP1?
Papilloma				HPV E2 [47]; HPV E6 [58]	High risk E6: PIASy [63]			
Parvo					B19 NS1: PIAS3 [61]			
Polyoma								
Pox								VACV I? [117]
Arena								
Bunya					NP: PIAS1 [67]			
Calici								
Corona								
Flavi					HCV: PIAS3? [62]	JEV [69]		
Filo					EBOV VP35: PIAS1 [68]			
Orthomyxo	IAV/IBV: increased [33, 34]	IAV RNA pol [33]				IAV NP? [75]		
Paramyxo						HPIV? [78–80]		
Picorna						Entero? [78–80]	FMDV L^pro^ [84, 112]	
Reo								
Retro						HIV-1 NP [71–74]		HIV [115]
Rhabdo								
Toga								

*Viral Abbreviations*: *CELO* Chicken embryo lethal orphan, *ADV* adenovirus, *HSV* Herpes Simples virus, *HCMV* human cytomegalovirus, *HHV-6* human herpesvirus-6, *EBV* Epstein-Barr virus, *KSHV* Kaposi’s Sarcoma-associated herpesvirus, *HPV* human papilloma virus, *VACV* Vaccinia virus, *HCV* hepatitis C virus, *JEV* Japanese encephalitis virus, *EBOV* Ebola virus, *IAV* Influenza A virus, *IBV* Influenza B virus, *HPIV* human parainfluenza virus, *FMDV* foot-and-mouth disease virus, *HIV* human immunodeficiency virus;? – Probable manipulation

In contrast, some viruses benefit from increased protein sumoylation. Influenza virus (Type A and Type B; IAV and IBV) infection leads to a viral replication-dependent global increase in cellular sumoylation (Table 1) [33, 34]. While select influenza viral proteins are targeted for sumoylation [34], IAV infection substantially increased the modification of 76 cellular substrates by SUMO-1 and 117 cellular substrates by SUMO-2 [33]. This increase was paralleled by decreased sumoylation of over 500 cellular proteins [33], suggesting the exchange of SUMO-1/2/3 from pre-existing targets to a restricted set of new targets [33]. Furthermore, because increased gross SUMO conjugation was not observed following infection with multiple cytoplasmic-replicating RNA viruses, Domingues et al. propose that the induction of SUMO remodeling is a specific response to nuclear-replicating viruses [33]. Similarly, we documented a global increase in cellular sumoylation during EBV latency, which is mediated by the principal viral oncoprotein latent membrane protein-1 (LMP1; Table 1) [35]. LMP1-induced sumoylation of cellular proteins contributes to the oncogenic potential of LMP1 [35], modulation of innate immune responses [36], and the maintenance of latency [37], all of which suggest the importance of increased sumoylation during latent infections.

While understanding global changes in sumoylation processes is critical to understanding global cellular changes that occur during viral infection, it is also important to identify the changes in sumoylation of specific cellular targets to elucidate mechanisms by which viruses modulate cellular responses that are pathogenic and ensure their propagation. Deciphering how viruses manipulate components of the sumoylation machinery may lead to new therapeutic targets that inhibit sumoylation processes, thus inhibiting viral replication and pathogenesis.

### Viruses and *sumo/*SUMO levels

**Fig. 2:**
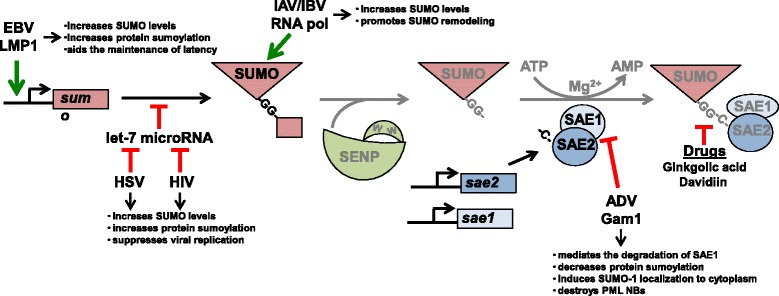
Targeting SUMO and the SUMO-activating Enzymes. Epstein-Barr virus (EBV) latent membrane protein 1 (LMP1) activates the *sumo* promoters, resulting in increased intracellular pools of SUMO and increased protein sumoylation. Following transcription of the *sumo* genes, let-7 microRNAs bind to the mRNA, inhibiting its translation. Herpes simplex virus (HSV) and HIV inhibit the expression of let-7 microRNAs, increasing protein sumoylation and suppressing viral replication. The influenza virus RNA polymerase also increases SUMO levels resulting in SUMO remodeling within the cell. Adenovirus (ADV) Gam1 induces the ubiquitination and degradation of SAE1. The loss of SAE1 promotes degradation of SAE2 and decreases the sumoylation of cellular proteins. Gam1 expression also induces the translocation of SUMO-1 to the cytoplasm and the destruction of the anti-viral promyelocytic leukemia (PML) nuclear bodies. The herbal ginkgolic acid and Davidiin both inhibit the interaction of SUMO with the SAEs

The first potential target of the SUMO machinery is the expression of the *sumo* genes. The four SUMO isoforms are found on different chromosomes, specifically chromosomes 2, 17, 21, and 6. To date, only the promoter for *sumo-1* has been identified [38], which has potential NF-κB, FOXP3, p53, and TCF-4E binding sites. While the transcriptional activity of these factors can be activated or repressed by a multitude of viruses and we have preliminary data that suggests the *sumo* promoters are activated during EBV latency through activation of NF-κB by LMP1 (unpublished data) (Fig. 2), the ability of any particular virus to activate the *sumo* promoters remains to be reported.

SUMO levels can also be regulated post-transcriptionally. For example, interferon-α treatment increases unconjugated SUMO-1 levels, but not *sumo-1* mRNA levels [29]. In this case, agonist binding to the toll-like receptors results in the activation of IFN and NF-κB signaling, which inhibits let-7 family microRNA levels and increases SUMO levels in treated cells [29]. This increase in SUMO levels suppresses HSV and HIV replication (Fig. 2) [29], highlighting one mechanism by which the sumoylation process mediates the antiviral effect of the interferons [29].

Influenza virus infection has also been shown to increase SUMO levels without increasing SUMO mRNA transcripts (Fig. 2) [33]. As explained above, IAV infection results in the sumoylation of nearly 200 cellular proteins, which is correlated with the de-sumoylation of over 500 cellular proteins [33]. While the distinct mechanism of the regulation of SUMO levels by influenza virus was not determined, it was shown to require the viral RNA, which led to the hypothesis that nuclear-replicating viruses trigger a stress response that induces SUMO remodeling and results in increased SUMO levels [33].

These findings identify three different mechanisms by which viruses can regulate *sumo*/SUMO levels following infection. First, the *sumo* promoters can be regulated by viral infection. Second, viruses can inhibit let-7 family microRNA levels to increase translation of *sumo* mRNAs. Third, viruses can regulate intracellular SUMO pools post-translationally by inducing SUMO remodeling. However, a better understanding of how intracellular *S*UMO levels are affected by viral infections is required to truly comprehend the function of sumoylation processes in viral replication and develop tools to manipulate protein sumoylation for therapeutic gains.

### Viruses and the SUMO-activating enzyme

The SUMO-activating enzyme is a common target for several sumoylation inhibitors, including ginkgolic acid (an alkylphenol from *Ginko biloba*) and Davidiin (an ellagitannin from *Davidia involucrata*) (Fig. 2) [39, 40]. These inhibitors bind to the SUMO-activating enzyme (SAE1/2) and impair the formation of the E1-SUMO intermediate [39, 40]. While there are additional cellular targets for these drugs, their effects on sumoylation processes have been documented [37, 39, 40]. Viruses have also evolved mechanisms by which they can impair the formation of the E1-SUMO intermediate. For example, infection of HeLa cells with avian adenovirus CELO (chicken embryo lethal orphan) induces a reduction of SAE1 and SAE2 (Fig. 2) [41, 42]. Mechanistically, this happens through the recruitment of the cullin RING ubiquitin ligases by the essential viral early protein Gam1 and the formation of a complex with SAE1/2 [42]. The cullin RING ubiquitin ligases ubiquitinate SAE1, resulting in the degradation of SAE1 by the proteasome [42]. The consequence of SAE1 degradation is an increase in unpaired SAE2, which leads to the subsequent proteasome-mediated degradation of SAE2 [42]. The end result is the accumulation of SUMO-unmodified substrates, increased localization of SUMO-1 in the cytoplasm, and destruction of PML nuclear bodies all of which contribute to enhanced viral propagation [43]. While the avian adenovirus is the only virus to date reported to target the SUMO-activating enzyme, it is highly likely that multiple viruses have the ability to inhibit, or possibly induce, the SUMO-activating enzyme, and thus the ability to modulate sumoylation processes.

### Viruses and Ubc9

The E2 SUMO-conjugating enzyme, Ubc9, has been proposed as an ideal target for therapies targeting the sumoylation pathway [44]. Two proposed methods of targeting Ubc9 include knockdown of Ubc9 levels by siRNA and over-expression of an enzymatically inactive Ubc9 (Ubc9 C93S) [44]. Recently the antibiotic Spectomycin B1 was documented to bind directly to Ubc9 and inhibit the formation of the Ubc9-SUMO intermediate [45]. Viruses have been shown to be able to manipulate Ubc9 through direct interactions, Ubc9 degradation, or altered Ubc9 localization in order to promote viral infectivity and viral pathogenesis.

**Fig. 3:**
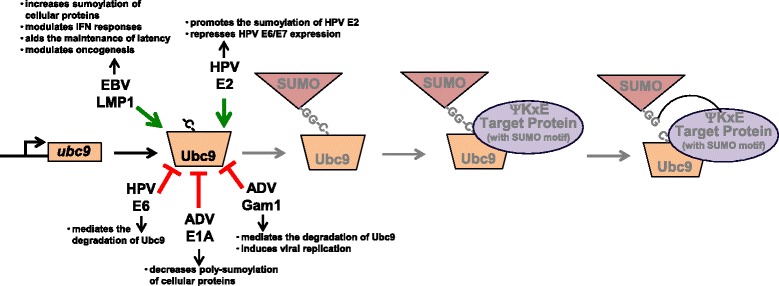
Targeting Ubc9. EBV LMP1 hijacks Ubc9 and increases the sumoylation of cellular proteins during latent infections, resulting in the maintenance of latency, oncogenesis, and modulated interferon responses. Human papilloma virus (HPV) E2 protein also hijacks Ubc9 to repress the expression of the viral oncogenes E6 and E7. Conversely, HPV E6 induces the degradation of Ubc9. ADV Gam1 increases the degradation of Ubc9, promoting viral replication. The ADV E1A also interacts with Ubc9 and blocks the poly-sumoylation of proteins

During the sumoylation process, potential target proteins interact with Ubc9. Some viruses are able to hijack Ubc9 for their own gain. The first viral gene expressed following human adenovirus infection (E1A) interacts with the N-terminus of Ubc9 (Fig. 3) [46], which results in competition between E1A and mono-sumoylated target proteins and inhibits the poly-sumoylation of the target proteins. While the direct Ubc9-interacting residues have not been mapped, EBV LMP1 also hijacks Ubc9 during latent viral infections (Fig. 3) [35]. We documented that the understudied C-terminal activating region 3 (CTAR3) was necessary and sufficient for this interaction [35]. While this interaction does not induce the sumoylation of LMP1 itself, the LMP1/Ubc9 interaction did result in increased sumoylation of other cellular proteins, which we subsequently showed to be important in modulating innate immune responses, maintaining viral latency, and the oncogenic potential of LMP1 [36, 37].

The human papillomavirus (HPV) protein (E2), which aids viral replication and genome segregation and down-regulates expression of the oncogenic E6 and E7, also interacts with Ubc9 (Fig. 3) [47]. However, instead of using this interaction to affect the sumoylation of other proteins, the viral E2 is itself sumoylated [47]. Inhibition of this sumoylation decreases the transcriptional activity of papillomavirus E2 and abrogated its repressive effects on E6/7 expression [47]. These findings suggests that the hijacking of Ubc9 by HPV E2 and the sumoylation of E2 has an inhibitory effect on viral promoters but an activating effect on select cellular promoters [47]. The ability of a viral protein to interact with Ubc9 and be sumoylated is not unique to HPV. Numerous viral proteins, including the immediate early herpesvirus proteins [48–56], have also been shown to interact with the SUMO conjugating enzyme and be sumoylated.

Independent of the enzymatic function of Ubc9 and the covalent attachment of SUMO to a target protein, human herpesvirus-6 immediate early protein 2 (IE2) interacts with Ubc9, which induces the repression of IE2-mediated promoter activation [57]. The viral IE2 lacks a consensus SUMO motif, and sumoylation of the viral protein has not been reported [57]. Instead, it appears that the viral IE2 hijacks Ubc9 in the nucleus to facilitate recruitment of a repressive transcription complex [57]; however, the role of this interaction in viral replication remains to be determined.

Instead of hijacking Ubc9 to increase protein sumoylation, some viruses induce the degradation of Ubc9. The HPV oncogenic protein E6 binds and leads to the degradation of Ubc9 by the proteasome (Fig. 3) [58]. E6-induced degradation of Ubc9 requires the cellular ubiquitin ligase E6AP [58]. Interestingly, Ubc9 levels increase during cervical lesion progression, suggesting the possibility of using Ubc9 levels to diagnose cervical cancer [59]. While these findings seem somewhat contradictory, it is possible that E6-induced degradation of Ubc9 is altered during cervical lesion progression, or as Heaton et al. propose, the reduction of Ubc9 may lead to subcellular region-specific substrate effects [58]. Similarly, the avian adenovirus early protein Gam1 interacts with Ubc9 (Fig. 3) [41]. While *ubc9* RNA levels were unaltered, Gam1 expression greatly reduced the stability of Ubc9 [41]. Gam1-induces proteasome-mediated degradation of Ubc9 did require an enzymatically active Ubc9 [41], suggesting there are unidentified sumoylation-specific aspects to this interaction. The end result of inhibition of sumoylation processes by Gam1 is the activation of transcription and the induction of an environment favorable for viral replication [41].

These data suggest that viruses have multiple mechanisms by which they can target the E2 SUMO-conjugating enzyme: they can hijack Ubc9 to regulate protein sumoylation; they can interact with Ubc9 to induce their own sumoylation and regulate their activity; they can interact with Ubc9 independent of cellular sumoylation processes; and they can induce the degradation of Ubc9 by the proteasome. Because Ubc9 has been suggested to be an ideal target for therapies targeting the sumoylation pathway [44], deciphering how specific viral proteins target the expression and function of Ubc9 may reveal potential interventions to treat different malignancies.

### Viruses and SUMO E3 ligases

**Fig. 4:**
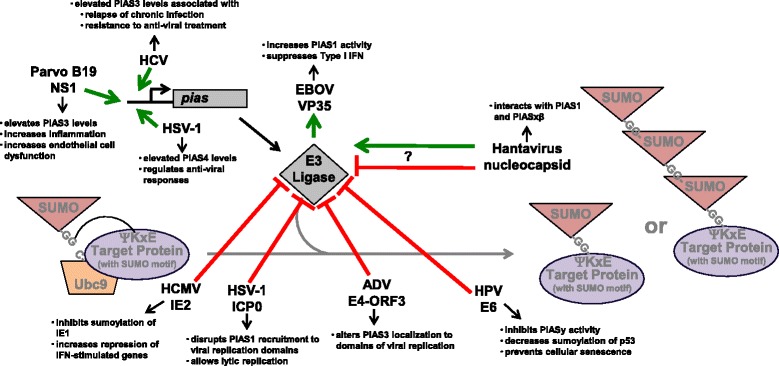
Targeting the SUMO E3 ligases (PIAS Family Members). HSV-1, Parvovirus B19, and possibly hepatitis C virus (HCV) induce the expression of PIAS family members resulting in regulation of inflammation and pathogenesis. ADV and HSV-1 alter the localization of PIAS family members to regulate viral replication. The Ebola virus (EBOV) VP35 protein increases PIAS1 activity to suppress immune responses and promote viral replication. The hantavirus nucleocapsid protein interacts with PIAS1 and PIASxβ, but the function for this interaction is known

While SUMO E3-ligases can guide substrate specificity, they are not required for sumoylation *in vitro*. Interestingly, the ability of viruses to manipulate SUMO E3 ligases, specifically the protein inhibitor of activated STATs (PIAS) family and RANBP2, has been investigated and reported more often than any other member of the SUMO machinery. Levels of members of the PIAS family are increased following some viral infections, resulting in dysregulation of antiviral immune responses. For example, PIAS4 levels are upregulated during HSV-1 infection (Fig. 4) [60]. During lytic replication PIAS4 is recruited to nuclear foci containing viral genomes and positively regulates intrinsic anti-viral immune responses to HSV infection [60]. Parvovirus B19 infection increases PIAS3 levels (Fig. 4) [61]. Specifically, the nonstructural viral protein NS1 transactivates numerous cellular promoters, including *pias3* [61]. PIAS3 can act as a negative regulator for STAT3, which is also phosphorylated and activated by NS1 [61]. The NS1-mediated modulation of STAT-targeted gene expression exacerbates inflammatory responses and results in endothelial cell dysfunction and viral pathogenesis, including viral cardiomyopathy [61]. In a similar correlation of PIAS3 levels and viral pathogenesis, increased PIAS3 levels are also associated with relapse of chronic hepatitis C virus infection and resistance to interferon-α treatment (Fig. 4) [62]; however, the mechanism for the events remain to be clarified. Overall, these reports suggest that some viruses induce PIAS expression in order to regulate viral replication and viral pathogenesis.

While some viruses induce the expression of SUMO E3 ligases, other viruses can inhibit cellular SUMO E3 ligase activity. For example, HPV E6 targets PIASy to inhibit the sumoylation of p53 and prevent cellular senescence (Fig. 4) [63]. Only E6 from high-risk papillomaviruses inhibited PIASy, which suggests that this selective targeting of the SUMO E3 ligases has a role in oncogenesis [63]. Similarly, human cytomegalovirus immediate-early protein (IE)-2 hijacks PIAS1 to inhibit the SUMO E3 ligase from sumoylating IE1 (Fig. 4) [50]. Decreased sumoylation of IE1 corresponded with enhanced repression of interferon-stimulated genes [50], which suggests the IE2-PIAS1 interaction is an important step in inhibiting inflammation and promoting viral replication.

Both adenoviruses and HSV-1 are thought to regulate the activity of SUMO E3 ligases by altering their localization (Fig. 4). First, the adenoviral E4-ORF3, which disrupts the anti-viral PML nuclear bodies (a process regulated by sumoylation), specifically targets PIAS3 to the nuclear scaffolds associated with viral genome replication domains [64], suggesting sumoylation processes are being redirected to aid viral replication. Second, the disruption of anti-viral PML nuclear bodies is mediated by HSV-1 ICP0 which, in addition to having E3 ubiquitin ligase activity, possess SUMO-targeted ubiquitin ligase properties that target sumoylated proteins for degradation [65]. While PIAS1 restricts viral replication, ICP0 disrupts the recruitment of PIAS1 to viral replication domains, allowing lytic replication to proceed [66]. PIAS1, along with PIASxβ, interacts with the nucleocapsid protein of hantaviruses (Fig. 4), specifically Seoul virus and Hantann virus [67]. Analysis of the hantavirus nucleocapsid protein sequence revealed a conserved sumoylation motif; however, sumoylation of the nucleocapsid protein remains to be documented [67]. While the direct function of this protein-protein interaction during hantavirus replication remains unknown, it may be similar to the ICP0/PIAS1 interaction in modulating antiviral responses and aiding viral replication.

In contrast to infection by HPV, adenoviruses, HSV, and hantaviruses, Ebola virus infection increases the activity of the SUMO E3 ligase PIAS1 (Fig. 4) [68]. The viral VP35 suppresses the production of type I interferons due to its interaction with interferon regulatory factors (IRF)-3 and −7 [68]. Ubc9 and PIAS1 are recruited to the VP35/IRF interaction, resulting in the sumoylation of these transcription factors and their transcriptional repression [68]. The modulatory effect that Ebola virus VP35 has on IRF7 is similar to our findings of the regulation of this transcription factor by EBV LMP1 [36]. This leads us to propose that LMP1 may also interact with PIAS1 or another SUMO E3 ligase to increase the sumoylation of select cellular proteins. However, this remains to be tested.

**Fig. 5:**
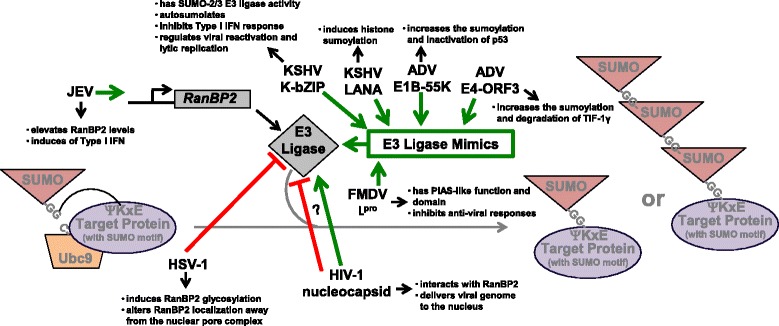
Targeting the SUMO E3 ligases (RanBP2 and SUMO E3 Ligase Mimics). Japanese encephalitis virus induces RanBP2 levels, which results in the activation of the Type I interferons. The HIV-1 nucleocapsid interacts with the E3 ligase RanBP2 to ensure the delivery of the viral genome to the nucleus. Both Kaposi’s sarcoma-associate herpesvirus (KSHV) and ADV encode viral E3 ligase mimics. While the KSHV mimic is specific for SUMO-2/3 conjugation, the ADV mimic specificity is unknown. FMDV L^pro^ has a PIAS-like domain and PIAS-like functions in order to inhibit anti-viral responses and allow viral replication to proceed

While there have been multiple investigations into the viral manipulation of PIAS family members, fewer studies have examined how viruses affect the nuclear pore protein RanBP2, which has SUMO E3 ligase activity [8]. RanBP2 levels are upregulated following Japanese encephalitis virus infection (Fig. 5) [69]. Knockdown of RanBP2 increases viral replication and decreases production of Type I interferons, which suggests that Japanese encephalitis virus-induced RanBP2 levels have an anti-viral function [69], limiting viral replication and viral-mediated immune responses.

Virus-induced manipulation of RanBP2 is thought to regulate the movement of proteins and viral genomes into and out of the nucleus. HSV infection induces the glycosylation of RanBP2 and reduces the interaction of RanBP2 with other members of the nuclear pore complex (Fig. 5) [70], leading to the proposal that glycosylated RanBP2 does not associate with the nuclear pores during HSV infection and alters the trafficking of proteins or complexes into and out of the nucleus [70]. During HIV-1 infection, RanBP2 is essential for the nuclear import of the viral genome (Fig. 4) [71, 72] by binding to the viral capsid (Fig. 5) [73]. Supporting the importance of RanBP2 to HIV replication is the finding that certain RanBP2 point mutations that map to the capsid-RanBP2 interacting domains have evolved in primates under positive selection [74]. While primates with these select RanBP2 mutations display enhanced HIV replication, the role of these mutations in the interaction of RanBP2 and the viral capsid and the nuclear import of viral genome is unknown.

Interestingly, a naturally occurring RanBP2 mutation has been associated with increased susceptibility to recurrent acute necrotizing encephalopathy following infection by influenza, HHV-6, Coxsackievirus, enteroviruses, or parainfluenza virus [75–81]. Similar to HIV infection, the naturally selected mutation enhances viral replication, which suggests that RanBP2 also aids delivery of the influenza genome to the nucleus. While the role of RanBP2 in sumoylation processes during HIV and influenza virus infection remains to be investigated, it is likely that protein sumoylation and the nuclear import of viral genomes is positively affected by these naturally occurring mutations.

### Viral mimics of the SUMO E3 ligases

Perhaps the most novel mechanism by which a virus can regulate the function of a SUMO E3 ligase is by encoding its own ligase, which is the case for Kaposi’s sarcoma-associated herpesvirus (KSHV), adenovirus, and foot-and-mouth disease virus (Fig. 5) [82–84]. First, KSHV encodes the early gene K-bZIP (open reading frame K8 spliced to adjoin the ZIP domain) that belongs to the basic region-leucine zipper family of transcription factors [85]. Multiple functions have been identified for K-bZIP [85–107], including a role as a SUMO adaptor where it recruits Ubc9 to induce the sumoylation and transcriptional repression of specific viral promoters [92]. While this is similar to our work investigating the hijacking of Ubc9 by Epstein-Barr virus LMP1 [35], K-bZIP is unique in that it has been identified to have SUMO E3 ligase activity that is specific for SUMO-2/3, which suggests K-bZIP may have a role in mediating the poly-sumoylation of target proteins [82]. K-bZIP auto-sumoylates itself and catalyzes the sumoylation of its interacting proteins (ex. p53 and Rb) [82], which ultimately inhibits the activation of the Type I interferons and regulates KSHV reactivation and lytic replication [98, 107, 108]. During KSHV latency, the latency-associated nuclear antigen (LANA) also acts as a SUMO E3 ligase recruiting the SUMO-Ubc9 intermediate and inducing the sumoylation of cellular histones (Fig. 5) [56]. While the exact function of this viral SUMO E3 ligase activity is unclear, it is likely to be critical in the maintenance of viral latency similar to our results on EBV LMP1 [35]. These findings highlight the importance of sumoylation processes in both lytic and latent herpesvirus infections.

Like KSHV, adenoviruses also encode two SUMO E3 ligase mimics. The adenoviral protein E1B-55 K, which regulates late viral gene expression, can also function as a SUMO E3 ligase that specifically induces the sumoylation of p53 and its localization of PML nuclear bodies (Fig. 5) [83]. The end result is the inactivation of p53, which is then exported from the nucleus and targeted for degradation by the proteasome [83]. Recently the adenoviral early protein E4-ORF3 was shown to possess SUMO E3 ligase activity due to its ability to mediate the sumoylation of transcription intermediate factor (TIF)-1γ, which has a role in transcriptional regulation and DNA damage repair (Fig. 5) [109, 110]. E4-ORF3 specifically induces the modification of TIF-1γ by SUMO-3 and aids poly-SUMO chain elongation, eventually leading to its degradation by the proteasome [110]. The eventual degradation of proteins targeted for sumoylation by both E1B-55 K and E4-ORF3 may help promote early and late adenoviral gene expression, facilitating viral replication.

The SUMO E3 ligase mimic for foot-and-mouth disease virus is different from the DNA viral mimics in that it has been assigned a specific PIAS-like function (Fig. 5) [84]. The viral proteinase (leader protein; L^pro^) is a papain-like proteinase that auto-catalytically self-cleaves from the viral polyprotein [111]. L^pro^ inhibits anti-viral responses and helps the virus evade host immune responses [112, 113], in part due to its ability to act as a de-ubiquitinating enzyme [114]. Recently, L^pro^ was shown to have a domain with PIAS-associated function [112]. Mutation of the L^pro^ PIAS-like domain significantly inhibits viral replication and viral pathogenesis [84]. In addition mutation of the L^pro^ PIAS-like domain increased the production of virus-specific neutralizing antibodies [84]. While proteins involved with sumoylation processes were not specifically examined, analyses showed that when compared with the mutant virus, wild-type virus was able to upregulate expression of numerous proteins associated with post-translational modifications [84]. It is probable that L^pro^ can act as a SUMO E3 ligase, targeting proteins involved with anti-viral responses, in order to allow viral replication to proceed.

Together, these findings show that although the SUMO E3 ligases may not be required for sumoylation i*n vitro*, they have an important role in regulating viral replication, anti-viral responses, and viral pathogenesis. Therefore, in the future the identification of additional mechanisms by which viruses can regulate the expression and activity of SUMO E3 ligases or even mimic the ligases will contribute to our knowledge of viral pathogenesis and suggest novel interventions in the treatment of viral disease.

### Viruses and SUMO proteases

**Fig. 6:**
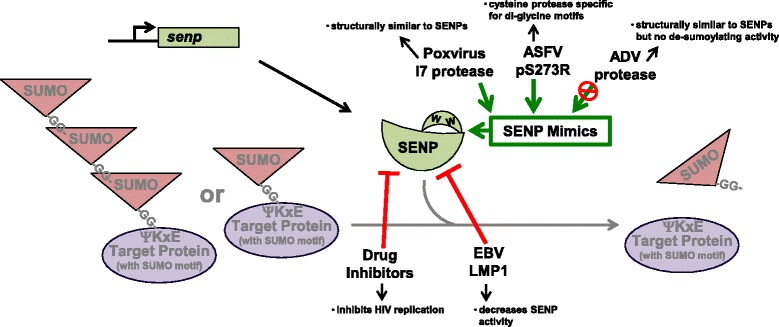
Targeting the SENPs. Poxviruses, African Swine Fever virus (ASFV) and ADV encode viral SENP mimics. Poxvirus I7 protease and the ADV protease are structurally similar to the SENPs. While I7 de-sumoylating activity has not yet been determined, the ADV protease lacks de-sumoylating activity. The ASFV cysteine proteins pS273R specifically cleaves following viral di-glycine motifs, but its ability to de-sumoylate cellular proteins remains unknown. EBV LMP1 inhibits cellular SENP activity, possibly by sumoylating the SENPs, which aids the accumulation of modified proteins during latency. Inhibition of SENP activity also functions to inhibit HIV replication

The SENPs regulate the intracellular pools of free SUMO as well as the de-sumoylation of modified target proteins, making them critical in the regulation of sumoylation processes. Interestingly, SENP inhibitors inhibit HIV replication [115], which suggests the SUMO proteases may be ideal targets for regulating viral infection or replication in vivo*.* Further, this finding also indicates that viruses have evolved mechanisms by which they use the SENPs in order to ensure viral dissemination*.* However, the effect viral infection has on SENPs remains to be documented. We have preliminary data suggesting that EBV LMP1 inhibits SENP activity during viral latency (unpublished data) (Fig. 6). When we specifically focused on SENP2, we found that LMP1 induced the sumoylation of SENP2, resulting in the inability of SENP2 to interact with sumoylated proteins, thus inhibiting its activity (unpublished data).

Just as some viruses encode their own SUMO E3 ligase, certain viruses are thought to encode SENP mimics. Vaccinia virus and fowlvirus encode a protease (I7) that is expressed late in infection (Fig. 6) [116]. The viral I7 protease has a C-terminal region with many structural similarities to the SENPs [117]; however the ability of this protease to actually cleave sumoylated proteins remains to be documented. African swine fever virus, a similar large, double-stranded DNA virus, encodes a cysteine protease (pS273R), which is a 31-kDa protein that has the conserved catalytic residues characteristic of SENPs [118]. pS273R specifically cleaves the viral polyproteins following a di-glycine motif (Fig. 6) [118], which coincides with the specificity of the SENPs [14]. Interestingly, S273R associates with the core of mature viral particles [118], suggesting a possibility that it, through its di-glycine motif-targeted cysteine protease activity, may have a function in the early steps following viral infection.

In some cases, the SENP mimics lack the ability to deconjugate sumoylated proteins. For example, the adenoviral protease processes viral proteins and has structural similarity to the *Saccharomyces cerevisiae* SUMO protease (Ulp1) [119]. While the adenoviral protease is essential for viral infectivity, it has been shown that it does not actually have the ability to de-sumoylate modified target proteins [119]; however, the possibility exists that the viral protease competes with the SENPs in interacting with sumoylated proteins. This could result in decreased SENP activity. Regardless, this study highlights the importance of elucidating functional targets of viral SENP mimics. Due to their role in regulating the maturation of the SUMO precursor and protein de-sumoylation [15], functional SENPs can affect target protein sumoylation and de-sumoylation. The critical role that dysregulation of cellular sumoylation processes has in viral infection, replication, and egress, suggests that SENPs may be an ideal viral target for manipulation of this cellular process.

### Other pathogens and manipulation of the SUMO machinery

The ability of a pathogen to manipulate sumoylation processes and members of the sumoylation machinery is not unique to just viruses of vertebrates. Sumoylation processes are important for White spot syndrome virus (WSSV) infection in crustaceans [120–123], where infection increases levels of SUMO and Ubc9 at the mRNA and protein levels [121, 122]. Silencing of SUMO and Ubc9 expression using RNA interference inhibits viral gene expression, viral replication, and shrimp mortality [122], which highlights the importance of sumoylation processes in the life cycle of WSSV. Another example is the geminivirus Rep protein [124], which binds to double-stranded DNA and catalyzes the cleavage and ligation of single-stranded DNA. Rep also hijacks the Ubc9 homolog in plants, increasing sumoylation processes, and aiding plant virus replication [124].

Outside of viruses, *Shigella spp.* also targets Ubc9 [125]. As a gram-negative bacterium, *Shigella* has a Type 3 secretion system that can deliver proteins into the cytosol of infected cells. As a result of the delivery of bacterial proteins to cells, *Shigella* decreases cellular Ubc9 levels along with the level of sumoylated proteins within the infected cell [125]. This suggests that one mechanism by which *Shigella* infection causes the pathology associated with shigellosis is through inhibition of cellular sumoylation processes.

## Conclusion

As we have outlined here, viruses possess multiple different mechanisms by which they manipulate the sumoylation machinery for their benefit and to the detriment of the host. Many investigations into sumoylation processes during viral infection have focused on the covalent modification of specific viral and cellular proteins. We have focused on how viruses affect the different steps of the sumoylation processes to regulate viral replication and viral pathogenesis. Viruses are able to target each step of the sumoylation process (Table 1), from the activation of the *sumo* promoters and altering the intracellular pools of free SUMO available for conjugation to the regulation of the expression/function of the SENPs.

Most previous research has focused on the manipulation of Ubc9 and the SUMO E3 ligases. However, as we have shown, there are additional possible targets by which viruses can influence sumoylation processes, and we have not addressed the role of SUMO-interacting motifs in the SUMO machinery and in viral proteins and SUMO-targeted ubiquitin ligases. Together, these findings highlight the importance of sumoylation processes in the viral life cycle and reveal the necessity of deciphering unknown mechanisms by which viruses target the cellular sumoylation machinery and sumoylation processes during their infection cycle.

The same pathway can be manipulated by different pathogens in different ways to achieve the same end goal, which is viral replication and propagation. Therefore, elucidating how viruses manipulate each step of the sumoylation process may reveal new targets for specific antiviral therapies. The viral-mediated targeting of the SUMO machinery to enhance/inhibit sumoylation processes could also have potential therapeutic effects in designing new treatments for cancer and other diseases where sumoylation processes are dysregulated.

## Abbreviations

ADV: Adenovirus
CELO: Chicken embryo lethal orphan
EBOV: Ebola virus
EBV: Epstein-barr virus
FMDV: Foot-and-mouth disease virus
HCMV: Human cytomegalovirus
HCV: Hepatitis C virus
HHV-6: Human herpesvirus-6
HIV: Human immunodeficiency virus
HPIV: Human parainfluenza virus
HPV: Human papilloma virus
HSV: herpes simples virus
IAV: Influenza A virus
IBV: Influenza B virus
ICP0: Infected cell protein 0
IE: Immediate early
JEV: Japanese encephalitis virus
KSHV: Kaposi’s sarcoma-associated herpesvirus
LANA: Latency-associated nuclear antigen
LMP1: Latent membrane protein-1
NP: Nucleocapsid protein
ORF: Open reading frame
PIAS: Protein inhibitor of activated STAT
RanBP2: Ran binding protein-2
VACV: Vaccinia virus
